# The formation of nanocrystalline ZrO_2_ nuclei in a Li2O-Al_2_O_3_-SiO_2_ glass – a combined XANES and TEM study

**DOI:** 10.1038/s41598-017-11228-7

**Published:** 2017-09-07

**Authors:** Enrico Kleebusch, Christian Patzig, Michael Krause, Yongfeng Hu, Thomas Höche, Christian Rüssel

**Affiliations:** 1 Otto Schott Institut, Jena University, Fraunhoferstraße 6, D-07743 Jena, Germany; 2 Fraunhofer Institute for Microstructure of Materials and Systems IMWS, Walter-Huelse-Straße 1, 06120 Halle (Saale), Germany; 3 Canadian Light Source Inc., University of Saskatchewan, 44 Innovation Boulevard, Saskatoon, S7N 2V3 Canada

## Abstract

The high economic importance of glass ceramics based on Li_2_O/Al_2_O_3_/SiO_2_ (LAS) is mainly due to their low coefficients of thermal expansion (CTE), which make these materials suitable candidates for a number of applications. The exact mechanism of the crystallization processes in LAS glasses is still not fully understood. The present work focuses on the formation and development of nanocrystalline ZrO_2_ within an LAS base composition which contains only ZrO_2_ as nucleating agent. Using a combination of transmission electron microscopy and X-ray absorption spectroscopy techniques, the temporal evolution of the ZrO_2_ nanocrystal formation is described. It is found that the formation of ZrO_2_ is initiated by liquid-liquid phase separation droplets with high Zr content, which eventually evolve into the nanocrystalline ZrO_2_ precipitations. This process is accompanied by a gradual change of the coordination of the tetravalent Zr ions from sixfold in the glass to eightfold in the crystals. The diameters of the ZrO_2_ crystals stay well below 4 nm, even at late stages. The degree of crystallization at each step of the crystallization process is deduced, and from that, the Avrami coefficient n is determined to be n ≈ 1, which describes a barrier-limited crystal growth process.

## Introduction

Nowadays, materials with low, i.e. near to zero thermal expansion find many applications, such as high temperature (furnace) windows, cook top panels, telescope mirrors, as well as numerous devices in optics and photonics^1^. Especially glass ceramics from the Li_2_O-Al_2_O_3_-SiO_2_ (LAS) system find widespread use in this respect^1^. The key physical property of these materials is the coefficient of thermal expansion (CTE), which should be close to zero over a wide range of temperatures^1^. This is essential especially for high temperature applications, because here, a high resistance against thermal shock is required, and thermal stresses in components with a strong temperature gradient are to be minimized^1^. In LAS glass ceramics, a CTE close to zero can be achieved if a high volume concentration of a crystalline phase with a negative thermal expansion coefficient is present in parallel to a host glass matrix that exhibits a positive thermal expansion coefficient. Besides this phase, other phases showing a positive coefficient of thermal expansion may also occur. By tailoring the volume fraction of the phases with different CTEs within the bulk LAS material, a CTE of the multi-phase glass ceramic material, that in total averages to zero, can be achieved^2, 3^. The crystalline LAS phases with negative thermal expansion that are suitable for low expansion glass ceramics are aluminosilicates, mainly β-spodumene, β-eucryptite, high-quartz solid solutions or keatite solid solutions^4–6^. Even if these glass ceramics exhibit a high volume fraction of the above-mentioned crystalline phases, they might nevertheless be fully transparent for visible light: if the crystal sizes are kept well-below half of the visible wavelength range, light scattering within the material is prevented. Thus, if transparent glass ceramics with a CTE close to zero are desired, care has to be taken that the crystal sizes do not extend some 10 nm^7, 8^.

A homogeneous bulk nucleation of numerous, nanoscaled LAS crystals with negative CTE is usually promoted by adding nucleating agents to the glass. Without the latter, surface nucleation will be the dominating crystallization process, leading to an (unwanted) fairly coarse, and - in surface-near regions – possibly even oriented microstructure. The addition of nucleating agents, however, may result in volume nucleation and crystallization of LAS crystals with sizes in the nanometer range, if the nucleation agent itself, once precipitated during thermal treatment of the glass, is homogeneously distributed within the glass bulk and has only nanoscale dimensions. Common nucleating agents for LAS glass ceramics described in the literature are TiO_2_^6, 9–13^ and ZrO_2_^14–17^, or both of them^14–19^, which commonly leads to the precipitation of ZrTiO_4_. We recently reported on the effect of the concentration of TiO_2_ and ZrO_2_ on the crystallization behaviour, especially on the resulting microstructure of the glass ceramics^19^.

The addition of nucleating agents to the LAS green glass composition is considered crucial to gain the aspired volume-crystallized LAS glass ceramics. Nevertheless, although a number of studies delivered considerable insights into aspects of nucleation in LAS glasses in recent years^20–24^, the complex mechanisms of the crystallisation of nucleation agents and the subsequent growth of the crystalline LAS phases are still not fully understood^25^. In the past, we have shown that it is possible to determine and describe the crystallization mechanism of the nucleation agent ZrTiO_4_ in LAS glass ceramics, with a combination of X-ray absorption near edge structure spectroscopy (XANES) and analytical transmission electron microscopy (TEM)^26^.

Only recently, we reported on the LAS phase formation and microstructure evolution as a function of the temperature and time of thermal treatment in glass ceramics that are solely nucleated with the nucleating agent ZrO_2_^27^. It was also reported that the ZrO_2_ concentration plays a crucial role in the systems MgO/Al_2_O_3_/SiO_2_ and MgO/ZnO/Al_2_O_3_/SiO_2_^28–32^.

In this paper, we go one step further and describe the crystallization behaviour of the nucleating agent itself within these LAS glasses, i.e., we describe the temporal course of crystallization of ZrO_2_ nanocrystals within a glassy matrix, at a given temperature of thermal treatment. By using a combination of X-ray Absorption Near Edge Structure spectroscopy (XANES), X-ray Diffraction (XRD), and (Scanning) Transmission Electron Microscopy (S)TEM including Energy-Dispersive X-Ray Spectroscopy (EDXS), the evolution of nanoscaled, Zr-rich liquid-liquid phase separation droplets into nanoscaled ZrO_2_ crystals is followed. For a given temperature, the fraction of Zr within the samples that is already crystallized to ZrO_2_ is derived as a function of the time. From the latter, the Avrami coefficient is deduced, and in combination with STEM-EDX results, it is shown that the mean diameter of the ZrO_2_ nanocrystals does not increase with time^27^, since they are surrounded by an Al-rich diffusion barrier^27^.

## Experimental

Raw materials of Li_2_CO_3_ (UCB), LiNO_3_ (Honeywell Riedel de Haën AG), Na_2_CO_3_, SiO_2_, K_2_CO_3_ (all from Carl Roth GmbH & Co. KG), 4 MgCO_3_·Mg(OH)_2_·4H_2_O (Merck KGaA), Al(OH)_3_ (Sumitomo Chemical), TiO_2_ (Germed DDR), ZnO (Vertriebsgemeinschaft für Harzer Zinkoxide GmbH (VHZ), Heubach), ZrO_2_ (Tosoh), Sb_2_O_3_ (Ferak Berlin GmbH) and BaCO_3_ (SABED) were used. The chemical composition of the studied glass is shown in Table 1. It is close to that of the commercially available Robax™ glass (SCHOTT AG), with the exception that as nucleating agent, only 3.0 mol% ZrO_2_ was chosen, while Robax™ contains both ZrO_2_ and TiO_2_.

**Table 1:** Chemical composition of the studied glass in mol%.

	Li_2_O	Na_2_O	K_2_O	MgO	BaO	ZnO	Al_2_O_3_	SiO_2_	TiO_2_	ZrO_2_	Sb_2_O_3_
**glass**	7.6	0.2	0.1	1.9	0.3	1.2	12.7	72.6	0.0	3.0	0.4

A batch of 300 g glass was melted in an inductively heated furnace within a platinum/rhodium crucible. First, a temperature of 1615 °C, kept for 2 h, was supplied. Then, the crucible with the melt was transferred to a MoSi_2_ furnace and for further melting, a temperature of 1680 °C was supplied and kept for 3 h. Finally, the glass melt was cast into a brass mold. The glass was then transferred to a muffle furnace, preheated to 700 °C. The furnace was subsequently switched off to allow the glass to cool to room temperature (cooling rate ~2 K/min).

The glass was cut into 0.5 × 0.5 × 0.5 cm^3^ pieces. To analyze the temporal course of the ZrO_2_ crystallization at a given temperature, a set of samples was thermally treated at 725 °C for different times *t*, ranging from *t* = 15 min to *t* = 24 h, in a pre-heated muffle furnace (Nabertherm).

For XRD investigations, the powdered samples were studied using a Rigaku MiniFlex300 X-Ray Diffractometer with Cu-*K*_α_ radiation (λ ≈ 0.154 nm) in a 2θ range from 10 to 60°.

The glass transition temperature was measured by dilatometry with a Netzsch Dil 402-PC dilatometer, using sample pieces with a length of 25 mm and a diameter of 8 mm (heating rate: 10 K/min). For differential scanning calorimetry, a Linseis DSC Pt-1600 calorimeter was used, supplying a heating rate of 10 K/min. The density was determined with a helium pycnometer (AccuPyc 1330).

X-ray absorption near edge spectroscopy (XANES) at the Zr *L*- edges was performed at the Canadian Light Source (CLS) in Saskatoon, SK, Canada. The experiments were run at the Soft X-ray Micro Characterization Beamline (SXRMB). As monochromator, a Si single crystal, cut in (111) direction, was used, resulting in a resolving power of 10^4^. The samples were fixed to the sample holder, using double-sided conducting carbon tape. The experiments were run in vacuum, with a residual pressure of approximately 10^−8^ mbar. The fluorescence yield (FY) data were recorded with a Si-Li drift detector. For the Zr *L*_2,3_-edges, the energy range between 2,215 and 2,330 eV was investigated. The FY data were normalized to the incident beam intensity (I_0_), and background corrected using the commercially available software UNIFIT 2014^33^, where an atan((*E*-*E*_*s*_)/*β*_*s*_) approach is used to describe the background at the edge jump, with the photon energy *E*, the edge position *E*_*s*_ and the FWHM of the step 2*β*_*s*_.

The micro- and nanostructure of chosen samples was further studied using (scanning) transmission electron microscopy ((S)TEM). The transmission electron microscopy analysis was performed with a *c*_*s*_-abberation corrected FEI TITAN^3^ 80–300 electron microscope at 80 kV acceleration voltage. The instrument is equipped with a high-angle annular dark field detector (Fischione Model 3000) to perform scanning TEM. In combination with energy-dispersive X-Ray spectroscopy analysis (EDXS) by means of a Super-X EDX detector that is equipped with four SDD detectors (FEI company), for some of the samples an element distribution analysis was performed. Element distribution mappings were obtained using the commercially available software *Esprit* (Bruker company). These mappings were derived by evaluation of the lateral distribution of the peak intensity, i.e., the area underlying the *K* edges of the analyzed elements, with an automatic, software-provided routine.

Sample preparation for (S)TEM was done using a purely mechanical wedge-polishing approach with a dedicated sample grinding and polishing tool (Multiprep, Allied company). Every sample was plane-parallel back-polished to a residual thickness of approximately 20 µm, followed by a second polishing step under a defined, small angle (2.6°) in order to render the samples into a wedge-like shape, whose edge tip is thin enough to be electron transparent. Each sample underwent a final Ar^+^ ion broad-beam milling step (precision ion polishing system PIPS, Gatan company) to remove polishing residues and to reach electron transparency. Finally, the samples were selectively carbon coated using a specific coating mask (CoatMaster, 3D- Micromac AG)^34^.

## Results and Discussion

The casted glass is colorless as an effect of the lack of titanium, since an ilmenite coloration due to Fe^3+^-O-Ti^4+^ charge transfer with trace impurities of iron, cannot take place^35^. As described in a further study, the T_g_ of the studied glass is determined on the basis of the appropriate DSC profile, and, moreover, with a dilatometric method^27^. It results in temperatures of 711 °C (DSC) and 703 °C (dilatometry). The density of the glass is 2.55 g/cm^3^ and the coefficient of thermal expansion (CTE) was found to be 4.60 × 10^−6^ K^−1^ in the temperature range from 100 to 500 °C.

As the focus of this study is on the temporal evolution of the nucleation agent ZrO_2_ within the LAS glasses, rather than on the subsequent LAS phase formation itself, several samples of the LAS glass with the composition as stated in Table 1 were thermally treated at 725 °C for several periods of time between *t* = 15 min and *t* = 24 h. A temperature of 725 °C was chosen, since previous XRD studies indicated that, if glasses of the same LAS composition as used here are thermally treated for 24 h at 720 °C, ZrO_2_ is precipitating, yet without the subsequent formation of LAS phases. On the other hand, after crystallization at 730 °C for 24 h, the crystallization of LAS is already indicated by XRD^27^. For the present study, it was tried to provide a temperature as high as possible to ensure an efficient crystallization of ZrO_2_. Nevertheless, the applied temperature should still be low enough to suppress the subsequent crystallization of the LAS phase, in order to monitor the crystallization of the nucleation agent within the LAS glass alone. Otherwise, the undisturbed crystallization of ZrO_2_ could potentially be affected by the onset of the subsequent crystallization of the LAS phase, which is, after all, promoted by the nucleation agent.

### XRD results

**Figure 1:**
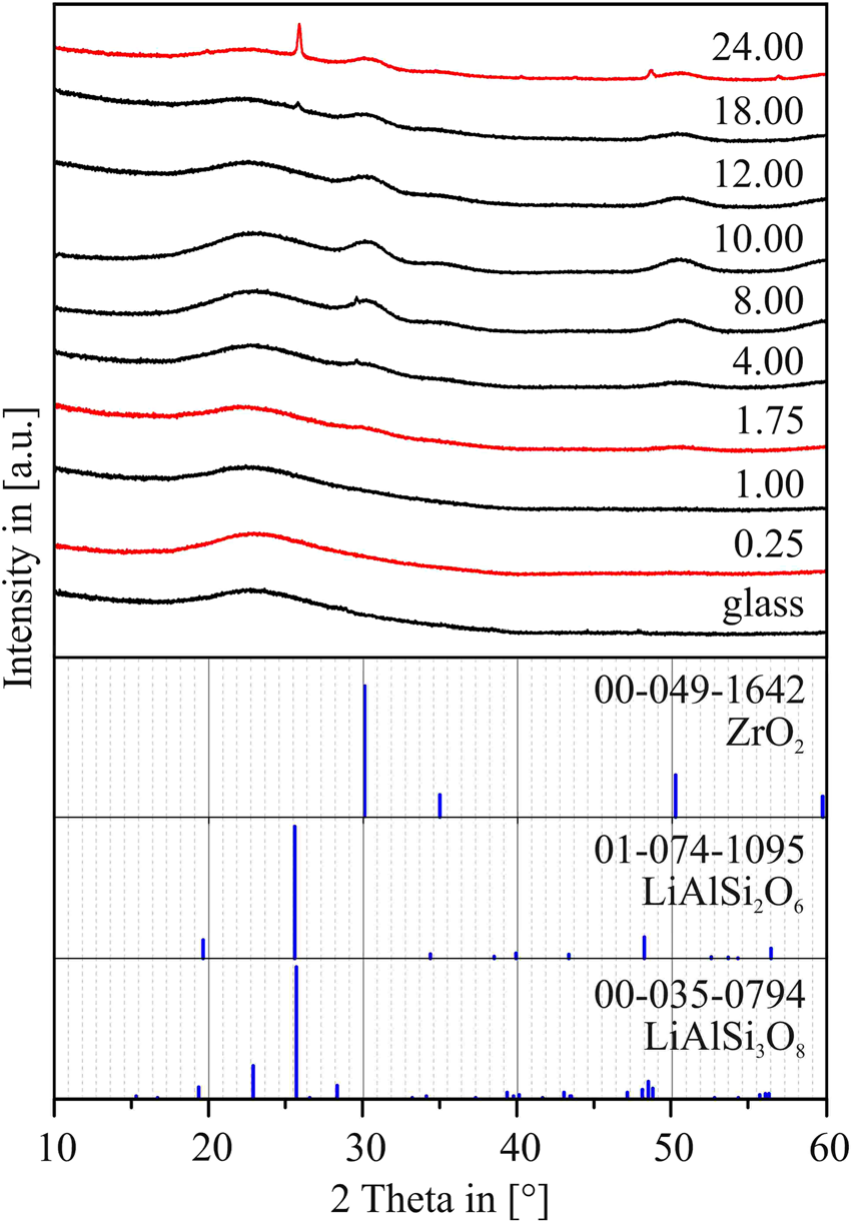
XRD patterns of LAS glass samples thermally treated at 725 °C for different periods of time [h]. The red highlighted patterns are described in detail in the discussion.

Figure 1 shows the XRD patterns of all samples, for which a constant crystallization temperature of 725 °C was supplied for different periods of times *t* (*t* = 0.25 h–24.00 h).

At the very early steps of nucleation, for short times *t*, no signs of the occurrence of crystalline phases within the samples can be found. The first visible, yet very notably broadened peaks at around 2θ = 30.1° and 52° can be observed after crystallization at *t* = 1.75 h, indicating the presence of a crystalline phase. These peaks can be attributed to either the tetragonal (JCPDS nr. 50–1089) or the cubic phase of ZrO_2_ (JCPDS nr. 49–1642). It is hard to distinguish between these two phases due to the only small tetragonal distortion, in combination with the XRD peak broadening due to the small crystallite sizes. After ongoing thermal treatment at *t* = 18 h, besides ZrO_2_, a low-intensity peak at 2θ = 25.6° also appears, which is the (101)-peak (100% peak) of the β-quartz LAS phase (JCPDS nr. 74–1095), i.e. the LAS phase which subsequently grows after initial nucleation of ZrO_2_ nanocrystals^27^. At *t* = 24 h, the intensity of the LAS peak increases notably, and is accompanied by further peaks at 2θ = 19.7, 48.5 and 56.8°, which are all attributable to the LAS β-quartz phase. Nevertheless, even at *t* = 24 h, the intensities of the LAS-related XRD peaks are still comparatively low, especially in relation to the broadened, main ZrO_2_ peak at 2θ = 30.1°. Thus, the XRD results as shown in Fig. 1 indicate the absence of any crystalline phase in the samples until a time of *t* ≈ 1.75 h is reached, where first signs of the appearance of crystalline ZrO_2_ can be seen. With ongoing heat treatment, the intensity of the ZrO_2_-related peaks increases, which is a clear sign for an increase of the quantity of crystalline ZrO_2_ within the samples. Finally, after *t* = 18 h, first LAS crystals occur in the sample – their quantity, however, is still quite low.

### TEM results

In order to illustrate the results obtained by XRD, electron transparent specimens were prepared from samples crystallized at 725 °C for *t* = 0.25, 1.75 and 24 h.

**Figure 2:**
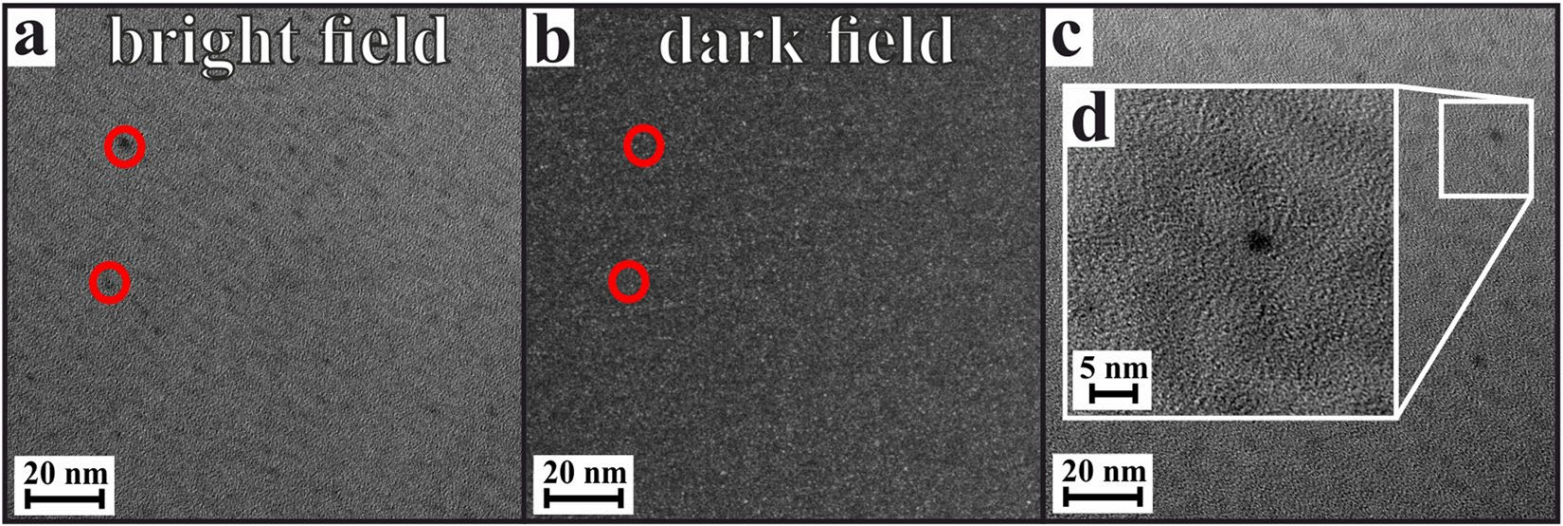
(**a**) TEM bright field micrograph, showing nanoscaled heterogeneities in the glass after *t* = 15 min at 725 °C. (**b**) TEM dark field micrograph of the sample sample area, showing no signs of crystallinity (**c**) TEM micrograph of nanoscaled heterogeneities at a different sample area and (**d**) detail of such a liquid-liquid phase separation droplet with a diameter of ≈2.5 nm.

Figure 2 shows TEM micrographs from a sample thermally treated for *t* = 0.25 h. In Fig. 2(a), the bright field micrograph clearly shows the occurrence of nanoscaled heterogeneities in the glass. These heterogeneities appear darker than the surrounding matrix, which indicates a larger density at these sample positions, as for TEM bright field microscopy, the direct electron beam is used for image formation, which is absorbed stronger in thicker or denser sample regions. However, these dense, nanoscaled inclusions within the glass matrix do not seem to be crystalline, since on the one hand, no lattice planes could be imaged at any of the analyzed sample positions. On the other hand, it was not possible to image these inclusions using TEM dark field imaging, which is a TEM imaging mode based on the use of diffracted electron beams for image formation: if a sample position is crystalline, and electron diffraction occurs due to the existence of a regular lattice plane arrangement in the crystals, this diffracted beam can be chosen for image formation using a selective aperture in the back focal plane of the TEM column. As it was not possible to image the dense inclusions that are present in the glass after *t* = 0.25 h that way, it may be concluded that these are indeed not crystalline, yet rather represent amorphous, liquid-liquid phase-separation droplets within the amorphous glass matrix. This finding is in agreement with the XRD patterns, which indicate the absence of any crystalline phases in the samples until *t* ≈ 1.75 h.

**Figure 3:**
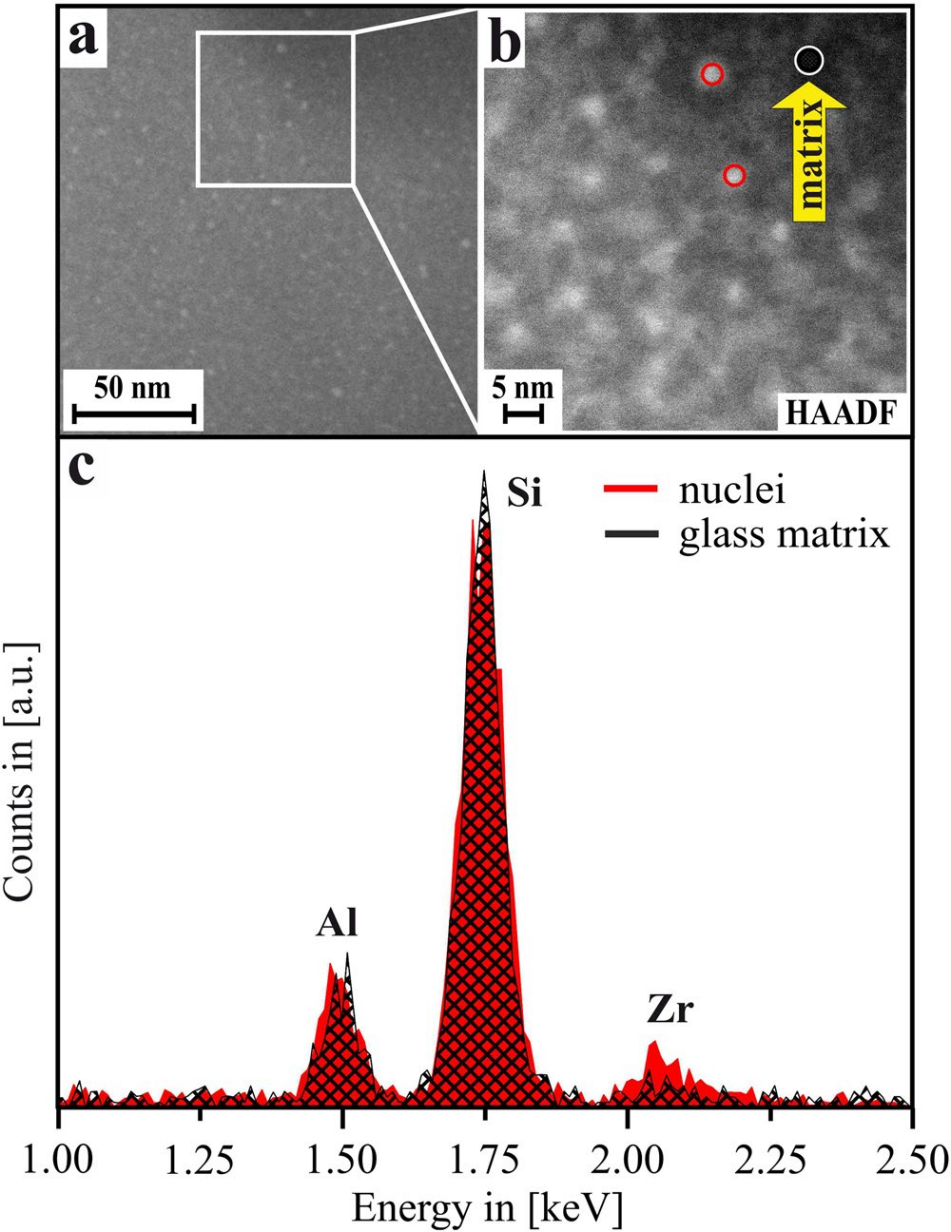
(**a**) STEM micrograph of a sample thermally treated at 725 °C for *t* = 0.25 h. (**b**) Detailed view of the inset shown in (**a**). (**c**) STEM-EDX spectra of different sample areas, as highlighted in (**b**).

Figure 3(a) and (b) show scanning TEM (STEM) micrographs of the same sample (*t* ≈ 1.75 h). As the image formation in STEM is based on inelastic scattering, in general it can be stated that dense or heavy sample positions appear brighter in the micrographs, and less-dense sample positions appear comparatively dark. Obviously, the STEM results clearly supply the TEM-based finding that dense, nanoscaled inclusions are dispersed throughout the glass matrix. As the STEM-EDX results in Fig. 3(c) show, these liquid-liquid phase-separation droplets are enriched in Zr, although Zr is still present within the surrounding matrix.

**Figure 4:**
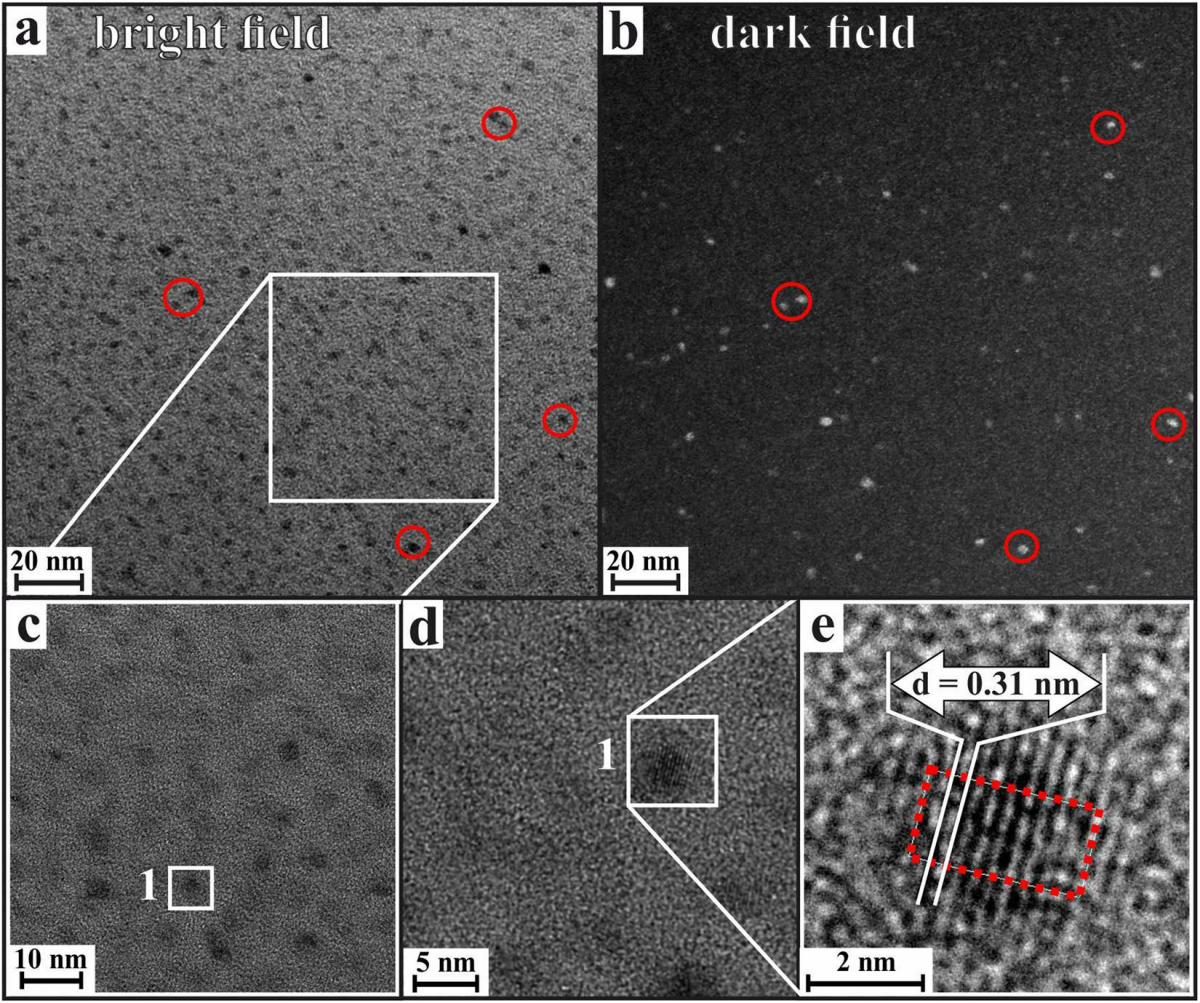
(**a**) TEM bright field micrograph of a LAS glass sample thermally treated at 725 °C for *t* = 1.75 h. (**b**) Same sample position: TEM dark field micrograph shows crystallinity of the nanoscaled inclusions. (**c**–**e**) Stepwise magnification of the inset in (**a**). The lattice plane distance correlates to the[011]-crystallographic plane of t-ZrO_2_.

After *t* = 1.75 h, according to the XRD results, the occurrence of ZrO_2_ crystals within the samples can be expected. Indeed, the TEM-based analysis of the nanostructure of a sample thermally treated for 1.75 h shows that the glass matrix incorporates nanocrystalline ZrO_2_ inclusions, as shown in Fig. 4. Based on software-based image processing^36^ of binarized dark field images, the average diameter of the ZrO_2_ crystals at this stage was determined as 3.4 ± 0.8 nm. On the other hand, according to these TEM results, it cannot be verified whether all of the dense inclusions within the glass matrix are already crystallized ZrO_2_, or if a number of these inclusions still resembles the state of liquid-liquid phase-separation droplets, which are evident especially at earlier stages of the temporal course of crystallization.

**Figure 5:**
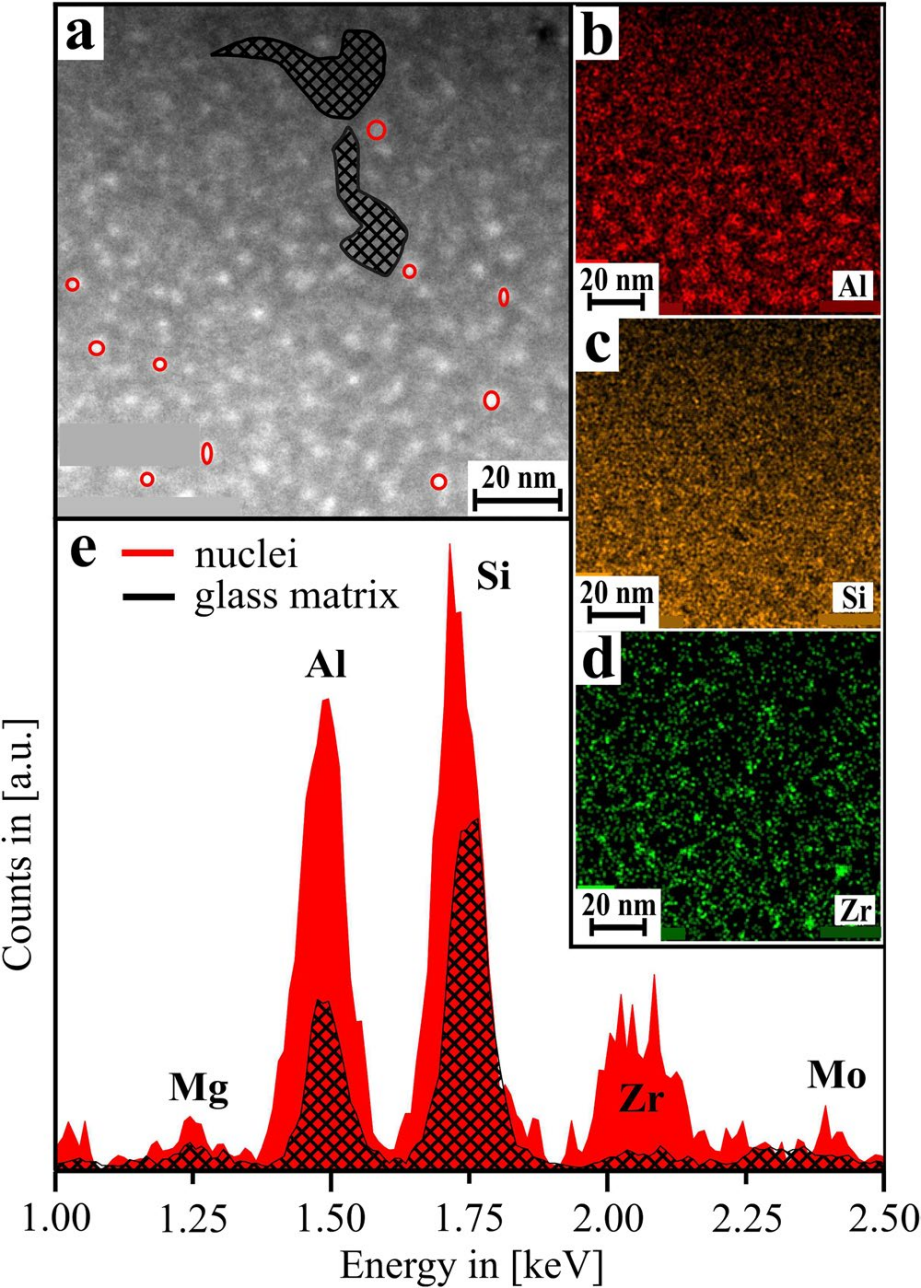
(**a**) STEM micrograph of a sample that was thermally treated at 725 °C for *t* = 1.75 h. (**b**–**d**) EDXS element distribution maps of the elements Al, Si and Zr, same sample area. (**f**) Red curve: accumulated EDX spectrum of single spectra gained via point measurements directly on the ZrO_2_ nanocrystals (highlighted as red circles in (**a**)); black curve: accumulated spectrum of the glassy sub-areas (highlighted as black hatched area in (**a**)). Mo is an artifact signal from the TEM experimental setup.

Figure 5 shows STEM micrographs and STEM-EDX results from the same sample at *t* = 1.75 h. Obviously, Zr is enriched at the positions of the droplet-like dense inclusions, which either already are ZrO_2_ crystals, or still are Zr-rich phase-separation droplets. However, as seen in Fig. 5(e), not only Zr, yet also Al is enriched at the positions of the Zr-rich inclusions: the Al/Si ratio at the sample positions that contain these nanoscaled, Zr-rich droplets is clearly larger than at the Zr-depleted glass matrix area. Since Al is not incorporated within the ZrO_2_ crystal lattice, this is an indication for an Al-rich shell that surrounds the ZrO_2_ nanocrystals at this stage – or, if the dense, droplet-like inclusion is not yet crystallized, it indicates that Al is most likely enriched within the droplet, and pushed towards the outer rim by the growing ZrO_2_ crystals once the crystallization thereof starts.

**Figure 6:**
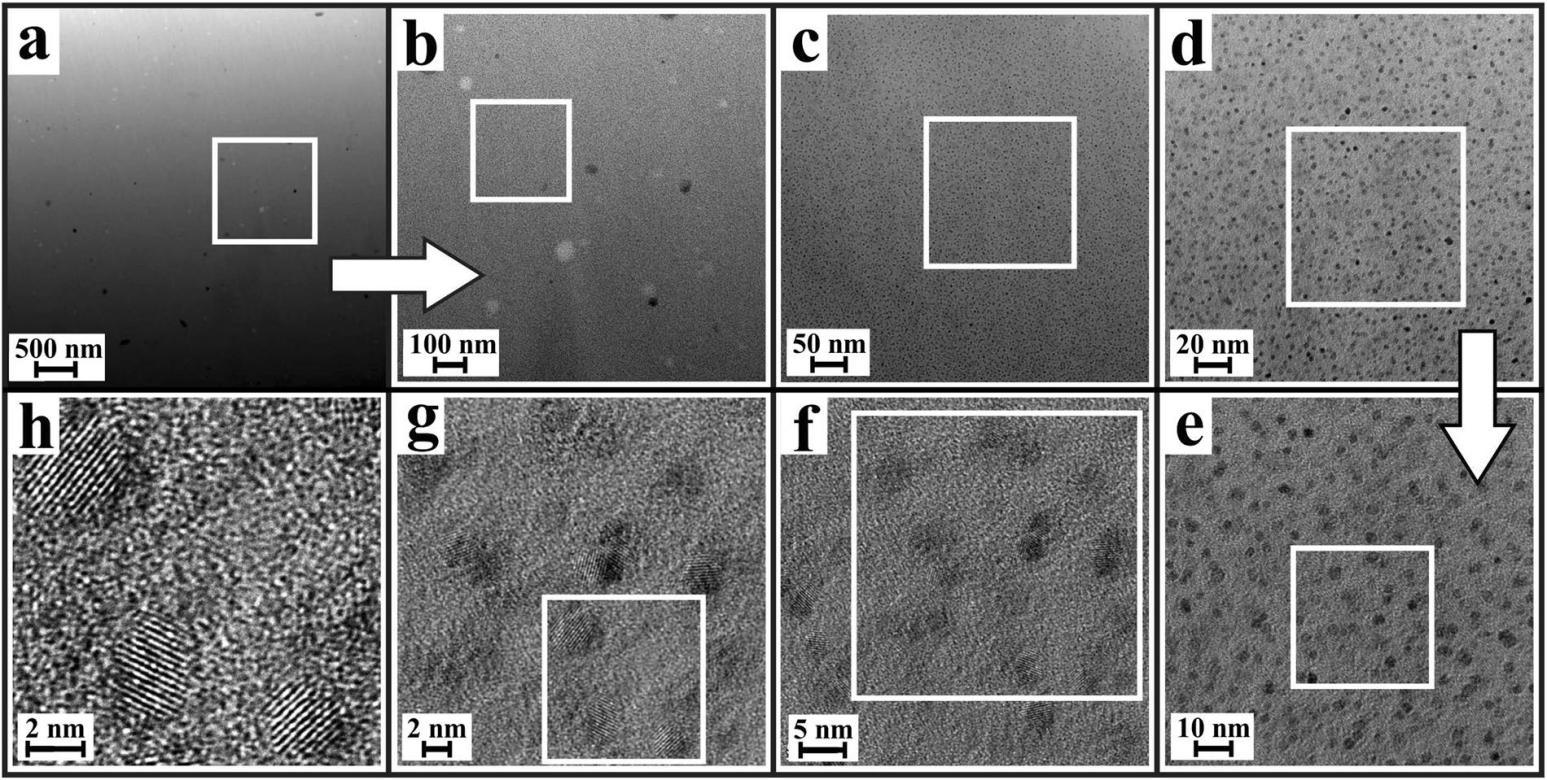
TEM bright field micrographs of a LAS glass sample thermally treated at 725 °C for *t* = 24 h. (**a**,**b**) At low magnifications, the LAS crystals with comparatively low volume density and crystal diameters of up to approximately 50 nm are visible. (**c**–**h**) With increasing magnification, the dense network of nanocrystalline ZrO_2_ precipitations gets visible.

Finally, after *t* = 24 h, according to XRD not only ZrO_2_, but also the LAS crystals should be present in the samples. Indeed, as seen in Fig. 6, TEM investigations of a sample from this nucleation stage show the occurrence of a few larger crystals with diameters of up to approximately 50 nm, as well as the expected dense network of ZrO_2_ nanocrystals. At this final stage, the average diameter of the ZrO_2_ nanocrystals is found to be 3.5 ± 0.7 nm. This means that the average size of the ZrO_2_ nanocrystals remains approximately constant all throughout the temporal course of crystallization, between the nucleation stage of the first appearance of crystalline ZrO_2_ at approximately *t* = 1.75 h and the final stage at *t* = 24 h, when first LAS crystals start to grow upon the ZrO_2_ nuclei.

**Figure 7:**
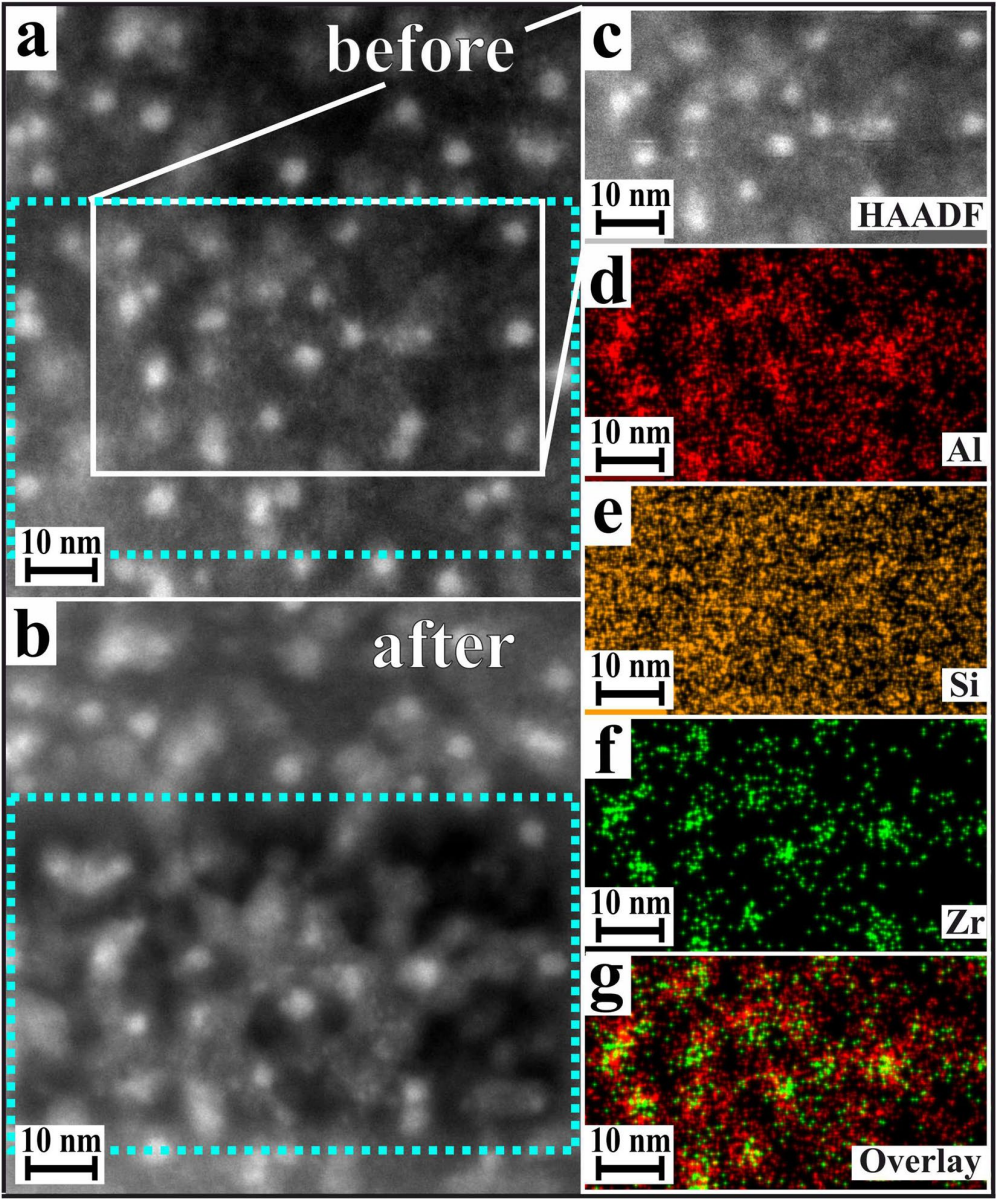
STEM-EDX mapping of a LAS glass sample thermally treated at 725 °C for *t* = 24 h. The comparison of the images (**a**,**b**) shows the radiation damage of the sample especially in the matrix area after the STEM-EDX investigations (highlighted by the blue dotted frame). The white frame in (**a**) shows the EDX-mapping area which is pointed out separately in (**c**). The images (**d**–**f**) show the enrichment of the elements Al, Si, and Zr. An overlay representation of the lateral distribution of Al and Zr is shown in (**g**).

Figure 7 shows STEM-EDX results of the same sample. Obviously, also after *t* = 24 h, an enrichment of Al close to the ZrO_2_ crystals appears.

### XANES results

**Figure 8:**
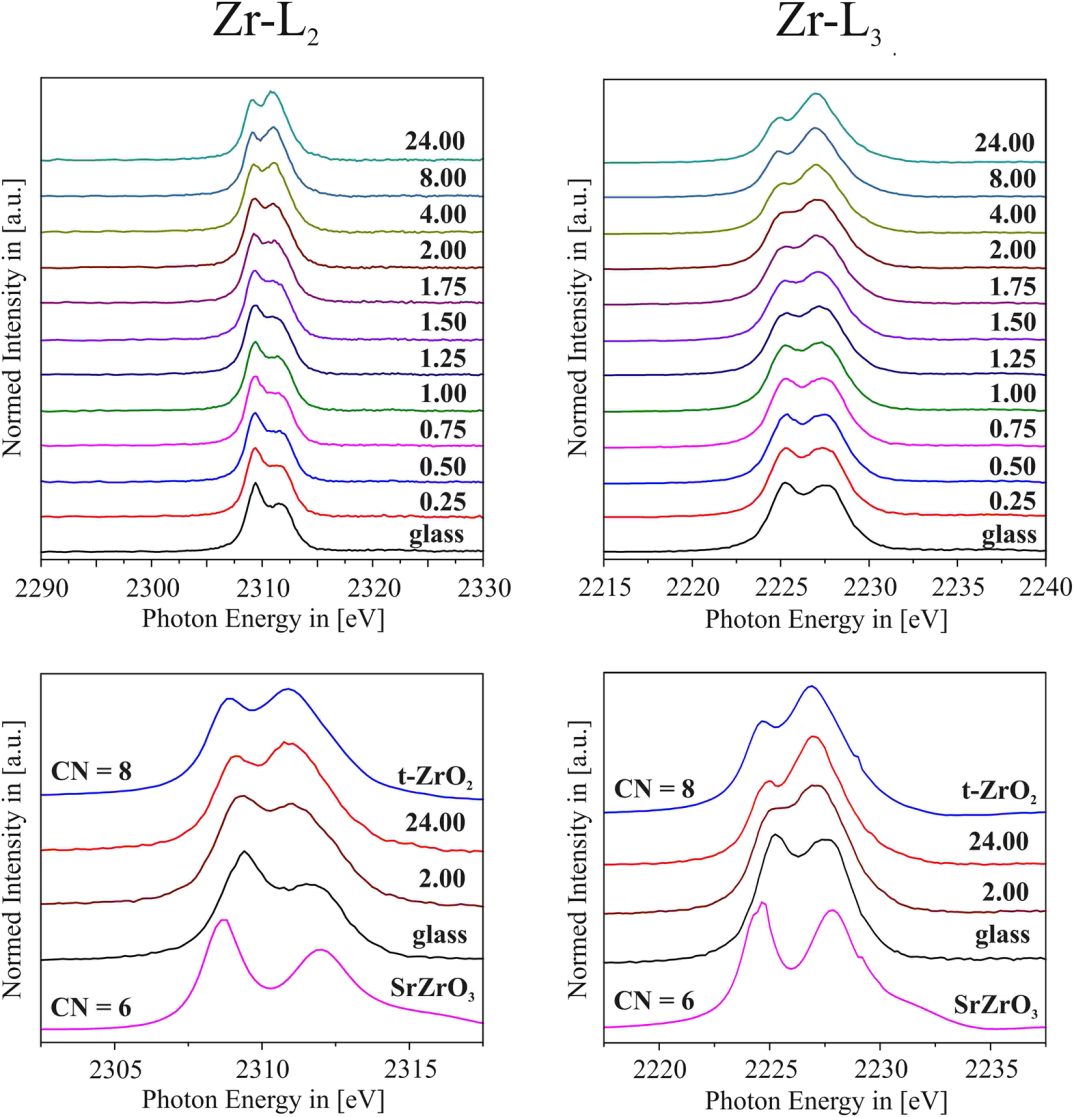
Top left and right: Summarized results of the Zr *L*_2_ and Zr *L*_3_ XANE spectra of samples heat treated at 725 °C for *t* = 0.25–24 h, showing the gradual change of the respective edges peak shape. Bottom left and right: Zr *L*_2_ and Zr *L*_3_ XANE spectra of reference materials that incorporate Zr^4+^ ions in sixfold (SrZrO_3_) and eightfold (t-ZrO_2_) coordination, and of the green glass sample as well of the samples thermally treated at 725 °C for *t* = 2 h and *t* = 24 h.

In the XANES spectra, the intensity ratios of the doublet peaks of both the Zr *L*_2_- and Zr *L*_3_- edges are sensitive to the coordination number of Zr and can therefore be used to estimate the coordination number (CN) of Zr by means of spectral fingerprinting^37^. In Fig. 8, it is shown that if the respective spectra of the LAS green glass and that of the sample at the final stage (725 °C, *t* = 24 h) are compared to reference spectra of Zr-containing compounds in which the tetravalent Zr^4+^ ions are either sixfold coordinated (^[6]^Zr^4+^ in SrZrO_3_), or eightfold coordinated (^[8]^Zr^4+^ in t-ZrO_2_), it is obvious that within the amorphous green glass matrix, the homogeneously distributed tetravalent Zr ions are mainly 6-fold coordinated. At the final stage, on the other hand, the shape of the Zr *L*_2_- and Zr *L*_*3*_- edge spectra and the intensity ratio of the respective doublet peaks rather resemble the spectra of the t-ZrO_2_ reference, showing that at this stage, the incorporation of Zr^4+^ ions into ZrO_2_ nanocrystals is basically completed. Thus, it seems reasonable to state that after this final stage at *t* = 24 h, indeed all of the previous liquid-liquid phase-separation droplets have evolved into nanoscaled ZrO_2_ crystals.

**Figure 9:**
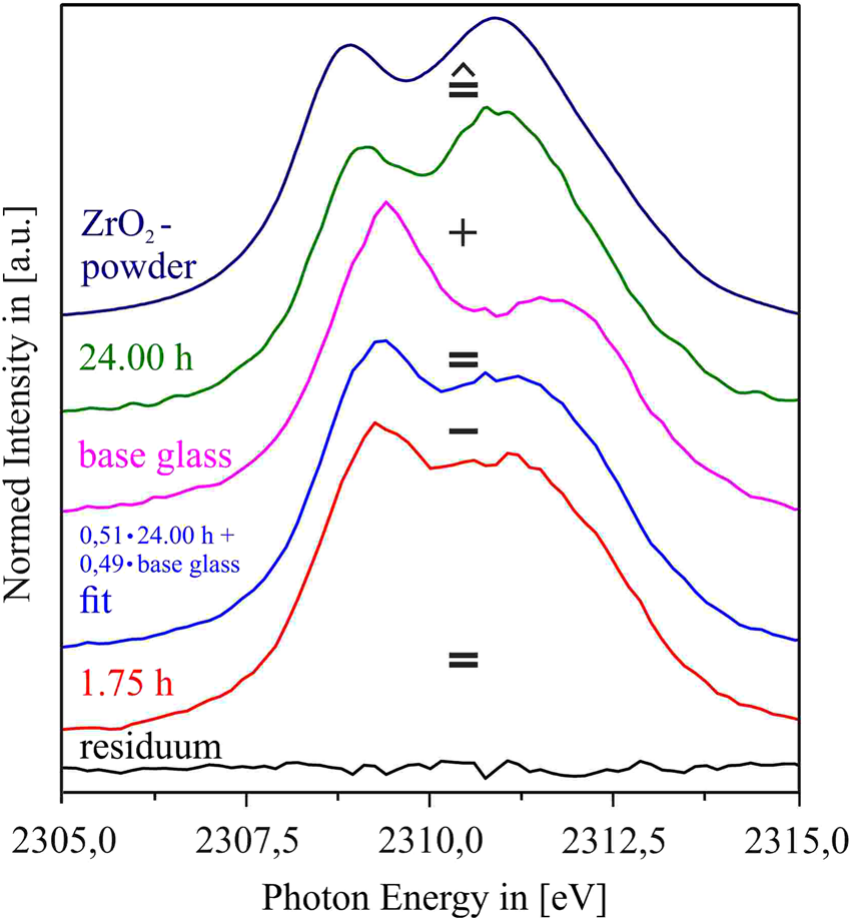
Schematic illustration of the linear combination of a weighted Zr *L*_2_ spectrum of the sample thermally treated at *t* = 24 h at 725 °C (representative for ^[8]^Zr^4+^ in fully crystalline environment) with that of a weighted Zr *L*_2_ spectrum of the green glass sample (representative for ^[6]^Zr^4+^ in fully amorphous environment) to gain a best fit of the recorded Zr *L*_2_ spectra of all time steps between green glass and *t* = 24 h (shown here: the best fit of the Zr *L*_2_ spectrum of the sample thermally treated for 1.75 h, which is gained by a combination of 0.51 * *green-glass-spectrum* + 0.49 * 24 *h-spectrum*, and the respective residuum, i.e., *fit – recorded data*).

The gradual change of the intensity ratios of the Zr *L*-edges as a function of time is shown in Fig. 8 as well. This change of the spectral shape is a very sensible way to probe the gradual transition of the coordination of Zr^4+^ ions from ^[6]^Zr^4+^ to ^[8]^Zr^4+^ within the LAS samples during the temporal evolution of ZrO_2_ crystallization^38^. In ref. 26, an approach for the Ti *L*_2*,3*_ XANES-based calculation of the degree of Ti^4+^ in crystalline environment in LAS glasses co-doped with ZrO_2_ and TiO_2_ is presented. In analogy, the degree of ZrO_2_ crystallization in the present study can be calculated as follows: at first, it has to be assumed that the crystallization of ZrO_2_ is finished after *t* = 24 h, meaning that all of the Zr that is present in the green glass composition is by then incorporated into nanocrystalline ZrO_2_. This assumption seems to be justified, since the Zr *L*_*2*_ spectrum of the sample that was thermally treated for *t* = 24 h shows a comparable doublet peak intensity ratio as the ZrO_2_ powder reference (cf. Fig. 9). Additionally, as Fig. 5 shows, there is nearly no Zr left in the glass matrix already after *t* = 1.75 h. Thus, between *t* = 1.75 h and *t* = 24 h, the Zr is either present in already crystallized ZrO_2_, or in Zr-rich liquid-liquid phase separation droplets that gradually evolve into nanoscaled ZrO_2_ crystals. As there are signs of a starting LAS crystallization after *t* = 24 h, it is very likely that the previous nucleation of ZrO_2_ is finished by then. Following this assumption, the Zr *L*_*2*_ spectrum of the *t* = 24 h sample is taken as ^[8]^Zr^4+^ reference spectrum, since for the present case, it appears more realistic to use the spectrum of a sample that contains ZrO_2_ nanocrystals embedded in a LAS matrix, rather than to use the spectrum of a polycrystalline, pure ZrO_2_ powder reference. For every time step, the respective Zr *L*_2_ spectrum is then simulated as a weighted linear superposition of a fraction α of the *t* = 24 h sample (that represents the final stage of ZrO_2_ crystallization, with only ^[8]^Zr^4+^ present in ZrO_2_ nanocrystals) and a fraction (1-α) of the green glass spectrum (that represents the spectrum for ^[6]^Zr^4+^ in amorphous environment, without any fraction of already crystallized ZrO_2_). The best fit for every time step is then determined by calculating the difference spectra of the α-dependent simulated spectra and the experimentally obtained spectra, and then by taking the sum of the squared difference spectra for all energies in the energy range of the Zr *L*_2_ edge, i.e. from 2,290–2,330 eV. For every time step, the best-fit α value is determined by the minimum of the sum of the squared difference spectra. Figure 9 exemplifies the procedure for the case of the *t* = 1.75 h sample: as can be seen, a linear superposition of (0.51 * green-glass-spectrum) + (0.49 * 24 *h*-spectrum) resembles the experimentally gained Zr *L*_2_ spectrum of the *t* = 1.75 h sample almost perfectly, with a residuum that is close to zero over the entire energy range. Thus, it can be concluded that the fraction α of ^[8]^Zr^4+^, i.e. of Zr^4+^ ions that already are nucleated into nanoscaled ZrO_2_ crystals, is approximately 51% after *t* = 1.75 h, meaning that half of all Zr present in the glass composition has turned into ZrO_2_ after that stage, while 49% of the Zr ions are still in an amorphous, sixfold coordinated environment. Since Fig. 5 shows that basically no Zr is left in the residual glass matrix after *t* = 1.75 h, this means that half of the Zr within the sample, which is sixfold coordinated, is present in form of the Zr-enriched, liquid-liquid phase separation droplets that have not yet been transformed into crystalline ZrO_2._ In other words, it can be concluded that at this stage of crystallization, half of the Zr-enriched droplets that are visible using TEM (cf. Figs 4 and 5) are nanocrystalline ZrO_2_, and the other half is still consisting of not-yet crystallized, Zr-rich phase-separation droplets.

**Figure 10:**
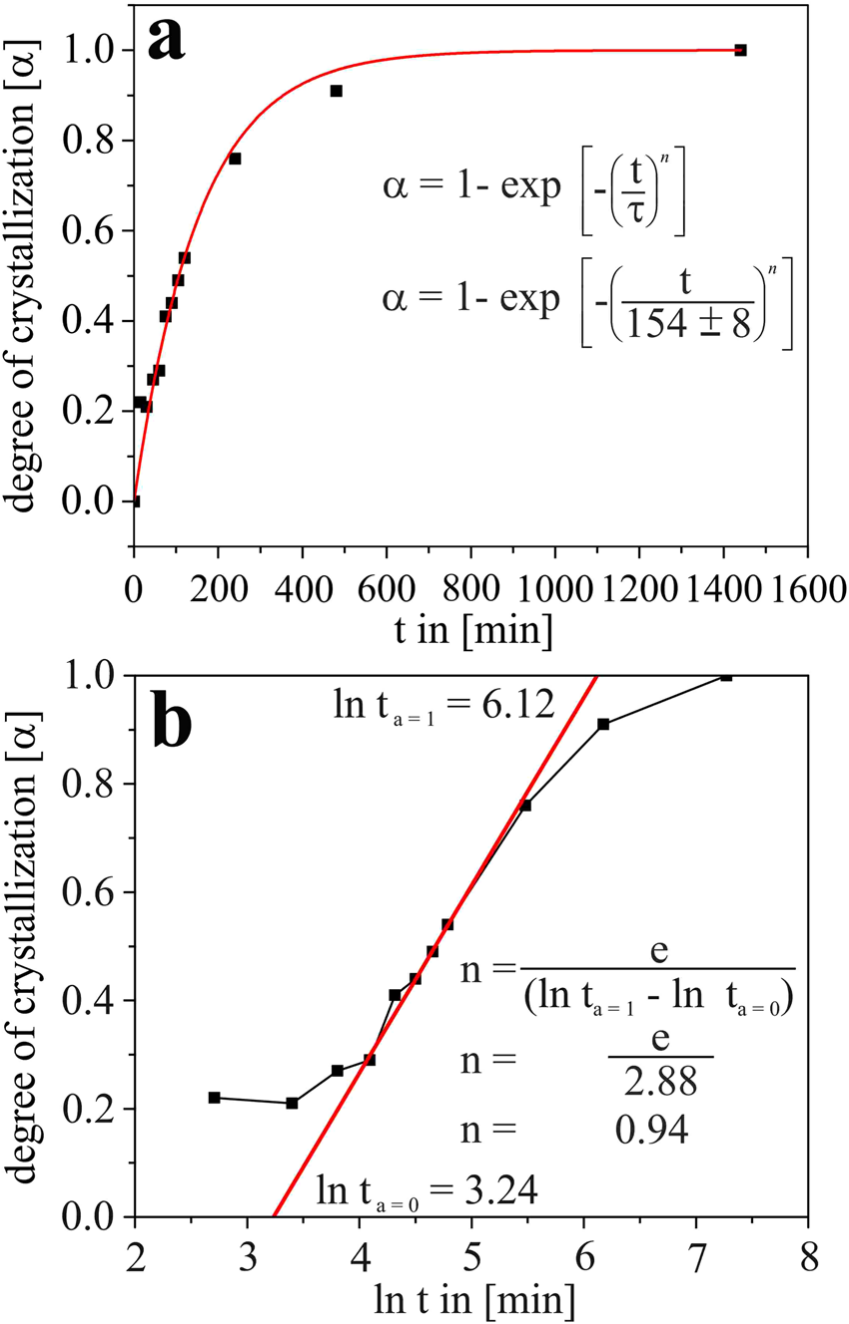
Degree of ZrO_2_ crystallization α after different times *t* of at 725 °C in (**a**) linear and (**b**) logarithmic representation. From (**b**), the Avrami coefficient of n = 0.94 ≈ 1 could be determined (for details, see text). In subfigure (**a**) the fitted Avrami plot with *n* set to 1 is shown as well.

Figure 10(a) shows the degree of Zr crystallization α as function of time of thermal treatment of the LAS glass at 725 °C. Obviously, the extrema of this curve are *α* = 0 for the initial glass (bearing no crystalline ZrO_2_) and *α* = 1 for the sample that was thermally treated for 24 h, from which it is assumed that all Zr of the glass composition is by then fully incorporated in ZrO_2_ crystals. Following that approach, which is further detailed in a previous publication^26^, this temporal evolution of α can be approximated with the Avrami equation:

1 $$\propto =1-\exp [-{(\frac{t}{\tau })}^{n}]$$

with *τ*: characteristic time and *n*: Avrami coefficient, whose value, which may range between 1 and 4, is specific for the crystallization mechanism that is taking place. As suggested in refs 26 and 39, *n* can be determined by plotting α versus ln *t* (see Fig. 10(b)) and determining the intersection of the straight line with the maximum possible slope of the resulting graph with the lines of α = 0 and α = 1. In our case, these values are ln t_*α* = 0_ = 3.24, and ln t_*α* = 1_ = 6.12. With these values, *n* can be calculated as follows:

2 $$n=\frac{e}{\mathrm{ln}\,{t}_{\propto =1}-\,\mathrm{ln}\,{t}_{\propto =2}}=0.94$$

This value for *n* close to *n* = 1 describes a barrier-limited crystal growth, where nucleation of ZrO_2_ is ever ongoing in the sample (as long as non-crystallized Zr is present), yet the newly formed crystals do not extend over a certain size as their growth stops at a barrier^26, 40–42^. Most likely, this barrier is defined by the Al enrichment that has been found near the ZrO_2_ crystals and Zr-rich devitrification droplets: the diffusion of Zr towards the crystallizing ZrO_2_ areas is hampered by this Al barrier, which eventually leads to a stop of the expansion of the single ZrO_2_ crystals within the glass matrix. This finding explains why the crystal size distribution of the ZrO_2_ crystals within the LAS samples thermally treated at 725 °C for several time steps is quite narrow and does not notably increase with time: as mentioned before, the average size of the ZrO_2_ crystals is approximately 3.5 nm at every crystallization step. No Ostwald ripening that would eventually yield larger ZrO_2_ crystals at the late stages takes place, since the growth of nuclei is literally frozen by an Al-rich barrier that surrounds the nanoscaled ZrO_2_ crystals.

The formation of core-shell like structures as a prerequisite for a narrow crystal size distribution was first reported in ref. 43. for an oxyfluoride glass composition. In contrast to the present investigation, there, the core-shell structure was not formed from a phase-separated state, but from a homogeneous glass. During the formation of CaF_2_ crystals within these oxyfluoride glasses, a diffusion layer, depleted in calcium and fluoride, and enriched in silica, is formed. It was argued that this layer increases in viscosity during crystal growth until a viscosity of 10^13^ dPa·s is reached – the layer then has a glass transition temperature equal to the crystallization temperature and further crystal growth is no longer possible.

The first stages of LAS crystallization is very different from that in the magnesium alumosilicate system. In the latter, comparatively large ZrO_2_ crystals are formed which do not exhibit spherical or cuboid shape. The tiny ZrO_2_ crystals, also observed in the MAS system are formed at the growth front of quartz during onward crystallization^44^.

## Conclusion

The present investigation reports on a glass ceramic of a selected chemical composition which is comparable to that of commercially available glass ceramic material Robax™. It was deliberately doped with ZrO_2_ as a single nucleating agent only, in order to nalyse the crystallization behaviour of this nucleating agent before subsequent LAS crystallization, especially in early stages of phase development.

At a given temperature of 725 °C, it was found that the crystallization of nanoscaled ZrO_2_ crystals with time takes place via the formation of Zr-rich liquid-liquid phase separation droplets, which eventually evolve into crystalline ZrO_2_. This transition is accompanied by a gradual change of the coordination of Zr^4+^ from sixfold (in the glass) to eightfold (in the ZrO_2_ crystals). It is found that the ZrO_2_ nanocrystals possess a narrow size distribution and do not notably increase their diameters over with time. This effect is linked to the occurrence of an Al-rich growth barrier that surrounds the ZrO_2_ crystals and most possibly prevents further diffusion of Zr ions from the glass toward the crystals, thereby freezing in their sizes to diameters of approximately 3.5 ± 0.7 nm, even at late stages.

The degree of crystallization at each stage *t* was determined, and from that, the Avrami coefficient was deduced to n ≈ 1, which describes a barrier-limited crystal growth and thus supports the assumption of the existence of an Al-rich growth barrier that surrounds the ZrO_2_ nanocrystals. It seems likely that the said barrier, whose chemical composition is strongly different from the green glass composition, enables ideal conditions for the subsequent crystallization of the LAS phases.
